# Echocardiographic assessment of aortic regurgitation: a practical guideline from the British Society of Echocardiography

**DOI:** 10.1186/s44156-024-00067-8

**Published:** 2025-01-27

**Authors:** Kelly Victor, Liam Ring, Vasiliki Tsampasian, David Oxborough, Sanjeev Bhattacharyya, Rebecca T. Hahn

**Affiliations:** 1 Cleveland Clinic London, 33 Grosvenor Place, London, SW1X 7HY UK; 2 West Suffolk Hospital NHS Foundation Trust, Bury St Edmunds, UK; 3 Norwich Medical School, University of East Anglia, Norwich, UK; 4 Cardiology Department, Norfolk and Norwich University Hospital, Norwich, UK; 5 Research Institute of Sports and Exercise Science and Liverpool Centre for Cardiovascular Science, Liverpool John Moores University, Liverpool, UK; 6 St Bartholomew’s Hospital, Bart’s Heart Centre, London, UK; 7 Columbia University Irving Medical Center, New York, USA

**Keywords:** Aortic regurgitation, Guideline, Echocardiography, Aortic insufficiency

## Abstract

Aortic regurgitation is the third most common valve lesion with increasing prevalence secondary to an ageing population. Transthoracic echocardiography plays a vital role in the identification and assessment of aortic regurgitation and proves essential in monitoring severity and determining the timing of intervention. Building on the foundations of previous British Society of Echocardiography (BSE) recommendations, this BSE guideline presents an update on how to approach an echocardiographic assessment of aortic regurgitation. It provides a practical, step-by-step guide to facilitate a comprehensive, high-quality echocardiographic assessment of aortic regurgitation. It discusses commonly encountered echocardiography-based challenges with suggestions regarding how this information is relevant in the interpretation and grading of regurgitation severity. Additionally, the value of other cardiac imaging modalities is discussed. The guideline concludes with an overview of aortic regurgitation in the clinical context, addressing chronic versus acute aortic regurgitation, which features prompt referral for intervention, and the consequences of combined valve disease.

## Introduction

Aortic regurgitation (AR) is the third most common valve lesion accounting for approximately 5% of adults undergoing intervention for severe valvular heart disease [1–4]. As with other heart valve diseases, AR is more frequently encountered in older individuals and therefore presents an increasing health burden with an ageing population. Echocardiography is central to the diagnosis, monitoring, and clinical decision-making of patients with AR. The current document is intended to ensure the delivery of high-quality echocardiography in the assessment of patients with AR and is an update to the previous British Society of Echocardiography (BSE) guidance published in 2012 [5]. The content within the guideline has been drawn from the available literature and evidence base [6–8]. This guide should be seen as supplementary to the BSE minimum dataset [9]. The intended benefit of this supplementary document is to:

- Support echocardiographers to perform a comprehensive standardised protocol for the assessment of AR in adult patients.
- Promote quality by defining an optimal methodology for the assessment of AR that is based on current evidence.
- Facilitate the accurate comparison of serial echocardiograms performed in patients at the same or different sites.
- Ensure echocardiography enables optimal management of patients with AR.

Table 1 outlines the sections in this guideline document.

**Table 1:** Subsections of the British Society of Echocardiography practical guide to aortic regurgitation

1. Anatomy
– Standard anatomy
– Variant anatomy of the aortic valve
2. Mechanism of aortic regurgitation
3. Echocardiographic assessment of chronic aortic regurgitation
– Qualitative parameters
– Semi-quantitative parameters
– Quantitative parameters
4. Additional key parameters in the assessment of chronic aortic regurgitation
– Left ventricular size and function
– Global longitudinal strain
– Aortic dimensions
5. Echocardiographic features that may prompt referral for intervention
6. Approach to the patient with chronic aortic regurgitation
7. Additional cardiac imaging
8. Acute aortic regurgitation
9. Combined valve disease
10. Suggested reporting template
11. Conclusion
12. References

## Anatomy

**Table:** 

**Key points**
• The aortic valve typically has three cusps but may also develop with one, two or four cusps
• Identifying variant anatomy such as a bicuspid aortic valve is useful as it provides information to the clinician regarding disease progression
• Patients with variant anatomy should have particular attention paid to aortic dimensions, should be enrolled in echocardiographic surveillance, and family screening should be offered

### Standard anatomy

The aortic valve (AV) is composed of the left ventricular outflow tract, aortic valve cusps, and aortic root (AoR) to the sinotubular junction (STJ) [10]. The AV typically has three semi-lunar cusps attached to the aortic wall, associated with outpouching or sinuses of Valsalva (SoV). When the curved path of the aortic cusp insertion points are tracked, the three-dimensional spatial configuration of the AV resembles a crown [11]. The most distal point of attachment of the cusps is the STJ. The free edges of the cusps overlap resulting in coaptation to ensure competence however fenestrations may occur as part of the ageing process. Because of the curved configuration of the cusps, fibrous intercusp triangles or trigones fill the space between leaflet attachment to the aorta (Ao); the membranous septum underlies the right intercusp triangle (between the right and non-coronary cusps). The fibrous interleaflet trigones function to allow dynamic changes in the size and shape of the AoR during the cardiac cycle [12].

**Fig. 1:**
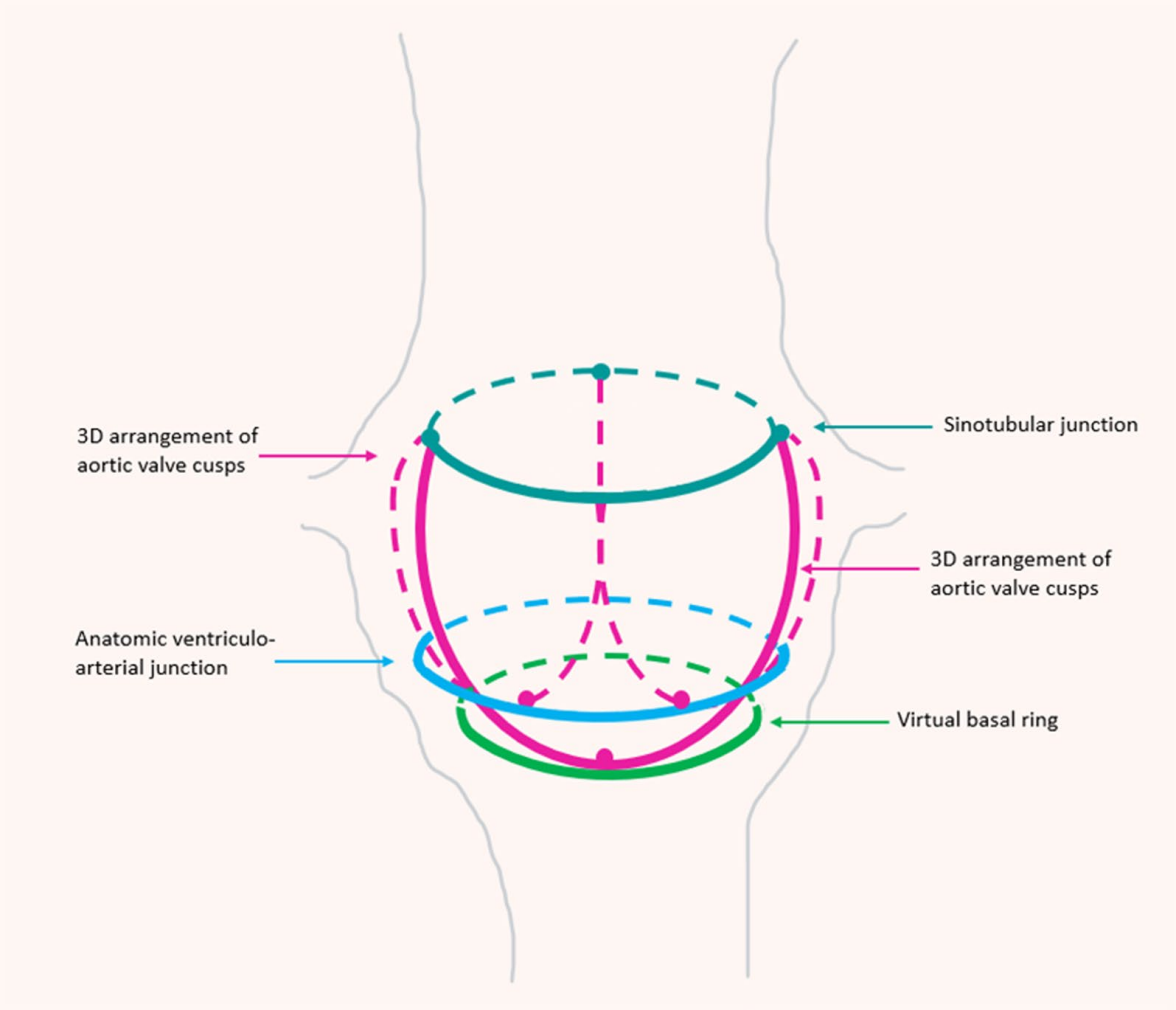
Graphical representation of the aortic annulus. Formed by the distal nadir the valve cusps, the virtual basal ring (green) is commonly referred to as the ‘aortic annulus’. Image adapted from Bleakley and Monaghan [18]

Although strictly speaking there is no ‘anatomic’ annulus, the most distal nadir of the three cusps defines a plane which is commonly referred to as the aortic annulus (Fig. 1) [8, 13, 14]. Two thirds of the circumference of the AoR is attached to the interventricular septum with the remaining third forming a fibrous continuity with the aorto-mitral curtain. The Ao itself is made up of three layers including the tunica intima, tunica media and tunica adventitia, and divided into anatomical segments: the sinus, incorporating the SoV, the STJ, and the tubular aorta. In normal anatomy, the left and right sinus give rise to the respective coronary artery ostia, with the posterior sinus a non-coronary sinus, and aptly named as such [15]. The three sinuses are positioned between the 'aortic annulus' and the STJ or ridge. The billowing shape of the sinuses facilitates AV opening in systole without obstruction to the coronary artery ostia [15]. The STJ is a tubular structure at the juncture between the commissures and the ascending aorta (AscAo), just distal to the SoV. It separates the AoR from the AscAo [16]. The AscAo is known as the tubular AscAo, beginning at the STJ and extending to the insertion point of the innominate artery within the aortic arch [17]. Normal anatomy of the AV and AoR using echocardiography is depicted in Table 2 [13].

**Table 2:**
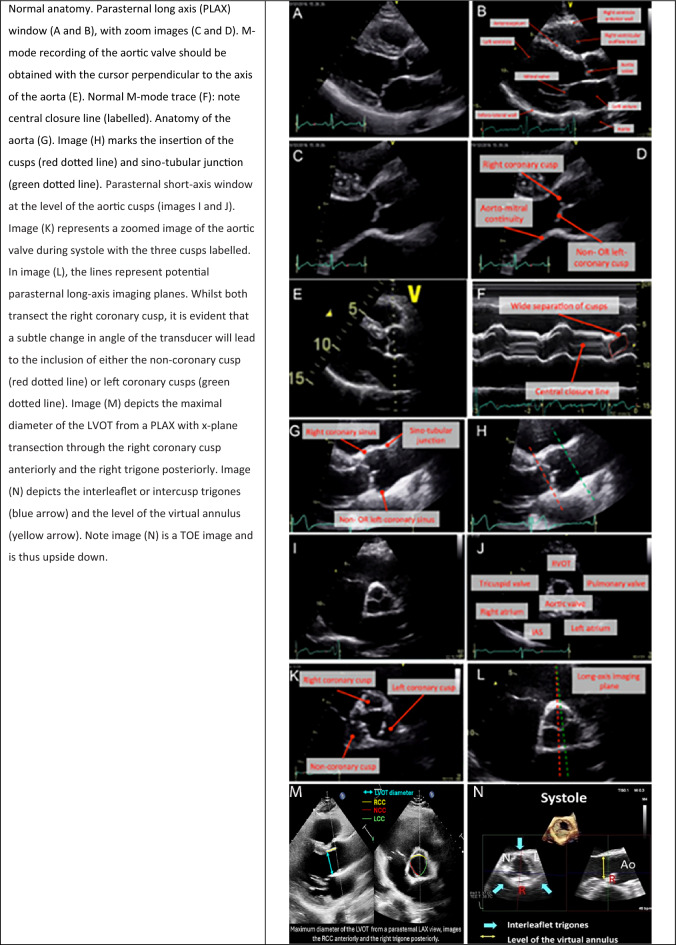
Normal anatomy of the aortic valve and aortic root using echocardiography [13]. Reproduced with permission from Ring et al.

### Variant anatomy of the aortic valve

**Fig. 2:**
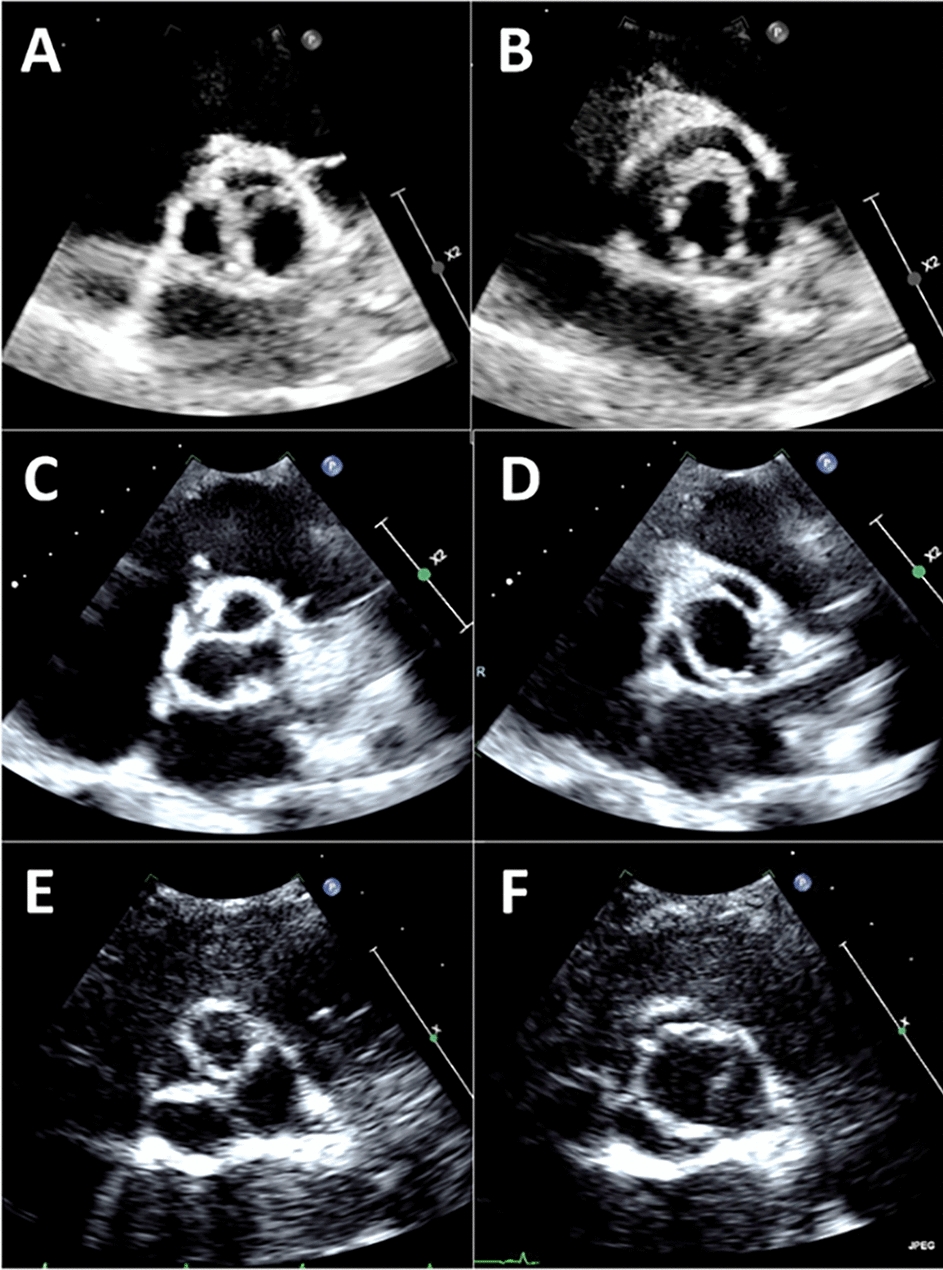
Variant anatomy of the aortic valve (AV); From a parasternal short axis, images (A) and (B) show a unicuspid AV in diastole and systole; Images (C) and (D) show a bicuspid AV with a left–right orientation and a raphe at approximately 3 o’clock in diastole and systole; Images (E) and (F) show a quadricuspid AV in diastole and systole

The AV may also develop with one, two, or four cusps (Fig. 2). Bicuspid aortic valves (BAV) are the most common anatomical variant, with a prevalence of approximately 1%, and a familial preponderance [13, 19–21]. BAV are commonly associated with AoR and AscAo dilatation, premature valve dysfunction, and increased risk of aortic dissection [22]. Classifications for the assessment of BAV morphology are inconsistent, with similar terminology often being used to describe different anatomical variations. This guideline recommends that BAV **should not be described** by 'type'. Instead, BAV should be reported descriptively by valve orientation (anterior–posterior vs left–right), noting fusion of the cusps, presence or absence of a raphe, and the number and symmetry of the sinuses [23]. Unicuspid (UAV) and quadricuspid aortic valves (QAV) are rare, with an approximate prevalence of 0.02% and < 0.01% respectively. QAV is more often associated with aortic dilatation and AR whereas UAV usually presents with aortic stenosis [24, 25]. Dysfunctional AVs frequently develop marked calcification which may impede the ability to clarify anatomy. In such circumstances, alternative imaging should be considered to establish the morphology of the valves. Irrespective of the presence or absence of valve dysfunction, all patients with variant anatomy should undergo echocardiographic surveillance owing to the high risk of disease progression. Close attention should be paid to aortic dimensions, and family screening should be offered to first degree relatives.

## Mechanism of aortic regurgitation

**Table:** 

**Key points**
• Aortic regurgitation is the result of aortic cusp abnormality, aortic root dilatation, or a combination of the two
• In all cases, the mechanism of AR should be characterised descriptively

Aortic regurgitation (AR) is the result of aortic cusp abnormality, aortic root (AoR) dilatation, or a combination of the two [6]. It is useful to classify the mechanism of valve insufficiency into one of three subtypes: Type I AR is characterised by *normal* cusp motion within the context of aortic dilatation or cusp perforation; Type II describes *excessive* motion such as cusp prolapse; and Type III is the consequence of cusp *restriction* [6]. Examples of each Type are provided in Table 3. The functional classification is a useful tool that helps clinicians systematically evaluate the valve behaviour and may influence the type of intervention chosen for the valve. However when reporting, it may be necessary to describe the mechanism rather than just quote the functional classification type. This would ensure findings are effectively communicated, relatable and transferable across echocardiographer, referrer and receiver (i.e. normal cusp motion in the setting of a dilated aorta).

**Table 3:**
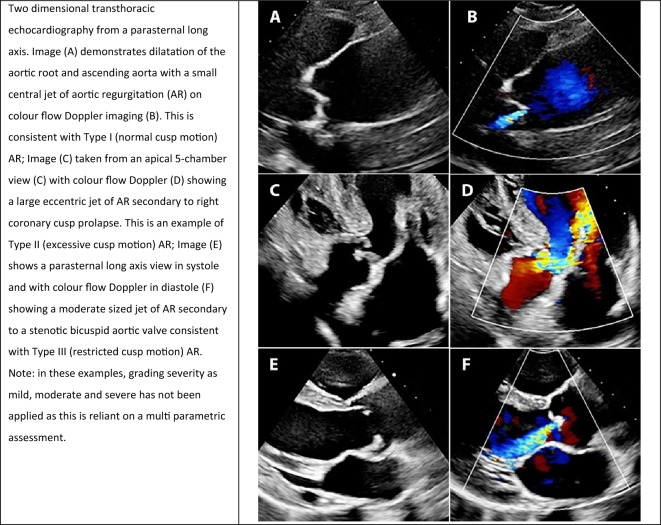
Examples of aortic regurgitation mechanism type according to the functional classification [6]

## Echocardiographic assessment of chronic aortic regurgitation

Within the following paragraphs, the key echocardiographic parameters central to a comprehensive assessment of AR will be described, along with a detailed explanation of how to obtain and optimise these measures.

In some circumstances, AR is obviously mild, in which case quantitative measures are not of use, will likely be challenging to obtain, and are of questionable accuracy. In such a scenario, confirming the mild nature of regurgitation using multiple acoustic windows and planes is sufficient. In all other circumstances, the British Society of Echocardiography (BSE) recommends that quantitative or semi-quantitative techniques are employed to assess the severity of AR and are coupled with corroborative findings (see ‘Approach to the patient’ section). It is important to appreciate that the following sections are focussed on the assessment of chronic AR with acute severe AR addressed later in the guideline.

Some general concepts for colour Doppler assessment of AR are worth noting:

1. It is important to visualise the three components of the colour jet (flow convergence, vena contracta and jet area) for a better assessment of the origin and direction of the jet, and its overall severity.
2. Colour Doppler jets are dependent on the systemic blood pressure (BP) but also ventricular compliance.
3. AR jets are frequently eccentric, moving in or out of the plane of view, constrained by the left ventricular outflow tract (LVOT), or entrained within the LVOT leading to rapid jet broadening.
4. Because of these variable characteristics, colour Doppler jet length or jet area from any window should not be used for assessing severity of AR.

### Qualitative parameters

#### Continuous wave spectral Doppler intensity

**Table:** 

**Key points**
• The use of continuous wave spectral Doppler intensity may provide a general impression of aortic regurgitation severity
• Given its inherent limitations and poor reliability, this technique should only be considered alongside other more rigorous parameters of severity

The intensity of the continuous wave (CW) spectral Doppler envelope offers a simple visual assessment that can be used to provide a general impression of AR severity. Technically, the density of the spectral Doppler signal is proportionate to the number of red blood cells travelling within the regurgitant jet: a weak or faint signal would indicate only mild regurgitation whereas a very dense signal is consistent with moderate or severe AR but is not able to conclusively differentiate the two. This method is unreliable with eccentric jets which may be transected by the alignment cursor and consequently underestimated (Table 4). Conversely, a narrow regurgitant jet well-aligned with the CW Doppler beam, may appear dense and overestimate the AR severity. Machine settings can also result in errors in assessment of severity. To limit errors, it may be advantageous to adjust the velocity scale to enhance the Doppler signal, and optimise the signal gain and reject to reduce transit-time artefact. Unfortunately, qualitative parameters, such as this one, are subject to inter and intra-operator variability further limiting their reliability. This guide recommends that CW Doppler intensity is only used as part of a multiparametric approach in the assessment of AR severity.

**Table 4:**
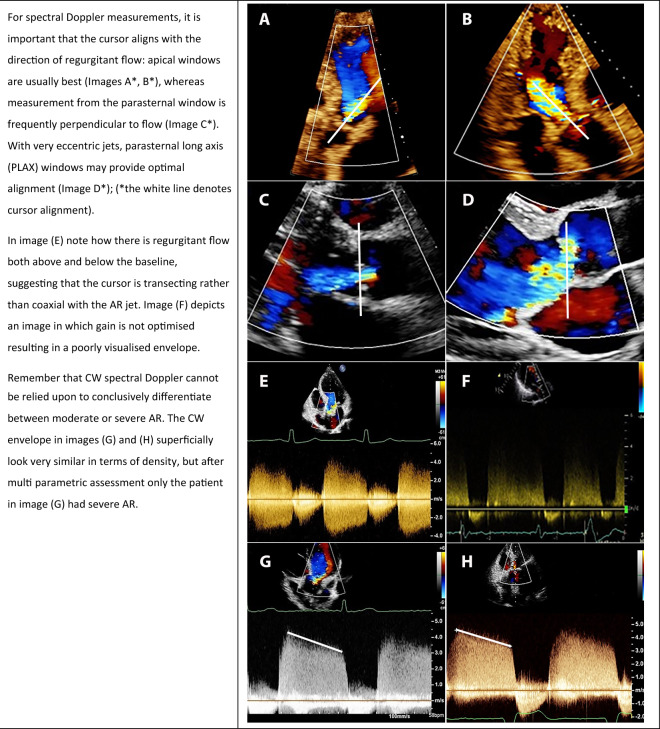
Spectral Doppler: how best to approach assessment of aortic regurgitation

#### Colour flow convergence zone

**Table:** 

**Key points**
• Colour flow convergence (CFC) is based on a qualitative, visual approach using multiple anatomical windows
• A small CFC hemisphere suggests mild aortic regurgitation while a large hemisphere visible throughout diastole suggests severe aortic regurgitation
• Jet momentum, jet direction, and orifice geometry are important considerations when evaluating CFC in the setting of aortic regurgitation severity

Proximal colour flow convergence (CFC) is a crude qualitative visual approach to the assessment of AR. It is based on the principle that a small hemisphere of CFC with small jet expansion corresponds with mild regurgitation, whereas a large hemisphere of CFC with large jet expansion predicts severe AR. There are several limitations associated with using this technique including jet momentum, jet direction, and orifice geometry which as a result of invalid hemispheric assumptions, may lead to under- or over-estimation. In the setting of confined flow convergence zones (such as perforation and commissural lesions) and obtuse flow convergence angles (such as cusp prolapse), assessment of AR severity using the CFC zone should be treated with caution [7]. When using this approach, there may be value in utilising a multi-plane function so that the CFC zone can be visualised in two orthogonal planes simultaneously (Table 5). Particularly for eccentric jets, multi-plane imaging may optimise the visualisation of the three components of the colour jet, essential for measurement of the CFC radius. For all jets, attempts should be made to slowly pan through the CFC zone so that it is captured at its maximum dimension and without hemisphere distortion due to coaxial imaging. Off axis imaging should also be considered where needed.

**Table 5:**
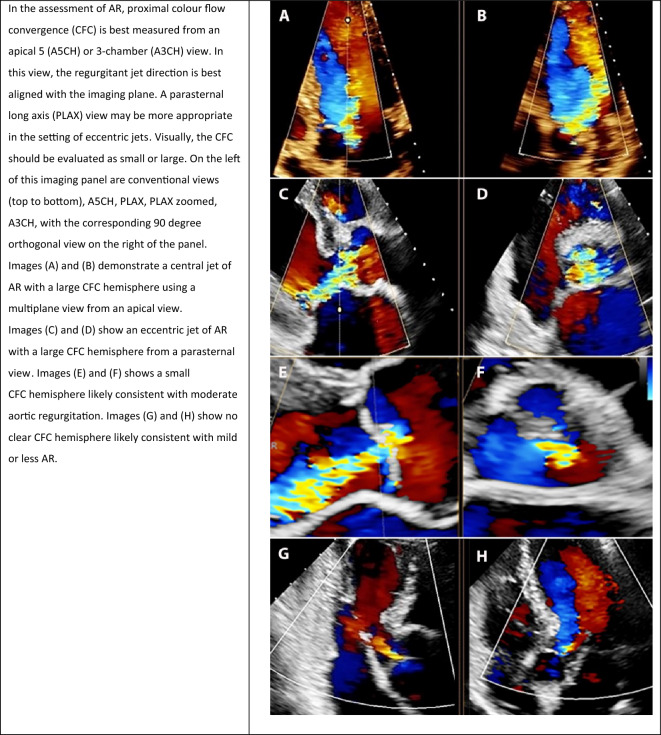
Multi-plane imaging of aortic regurgitation (AR): grading of AR severity based on colour flow convergence

#### Jet width/left ventricular outflow tract diameter ratio

**Table:** 

**Key points**
• Jet width / LVOTd ratio compares the colour flow jet width with the left ventricular outflow tract diameter
• A ratio of < 25% is consistent with mild aortic regurgitation
• A ratio of > 65% is consistent with severe aortic regurgitation

This is a simple measurement performed from the parasternal long axis (PLAX) window. However, there are three important limitations: (1) whilst measurement of the left ventricular outflow tract diameter (LVOTd) is optimised owing to excellent axial resolution, the colour Doppler measurement of the jet width may be underestimated depending on the jet angle; (2) a single view may not accurately characterise the three dimensional jet shape; (3) progressive jet broadening distal to the vena contracta limits reproducibility. Numerically, a jet width ratio of < 25% is consistent with mild AR, with a value of > 65% consistent with severe AR. Table 6 demonstrates the methodology for obtaining and optimising this parameter.

**Table 6:**
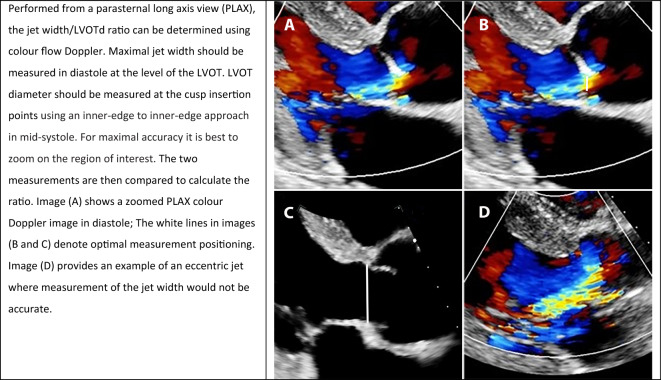
Jet width/left ventricular outflow tract diameter (LVOTd) ratio: methodology

#### Flow reversal in the descending thoracic aorta and abdominal aorta

**Table:** 

**Key points**
• Holodiastolic flow reversal within the descending thoracic aorta with an end-diastolic velocity ≥ 20cm/s is consistent with severe aortic regurgitation
• The presence of diastolic flow reversal in the abdominal aorta is consistent with severe aortic regurgitation
• Colour m-mode provides a graphical representation of reversal and can be used from both the suprasternal notch and the subcostal window
• Elastic recoil within the aorta may lead to ‘false positive’ findings of diastolic flow reversal

If the AR regurgitant volume is sufficiently large, blood flow in the aorta (Ao) will reverse during diastole. As AR becomes progressively more severe, diastolic flow reversal lasts proportionately more of diastole. Holodiastolic flow reversal with an end diastolic velocity ≥ 20cm/s in the descending thoracic Ao is both sensitive and highly specific for severe AR, whereas non-holodiastolic flow reversal is consistent with moderate AR (Table 7A). The presence of any flow reversal in the abdominal Ao, whilst not seen frequently, is a highly specific indicator for severe AR (Table 7B) [6, 26, 27]. Colour m-mode from the suprasternal notch (Table 7, (C)) and the subcostal view may prove helpful in the assessment of flow reversal duration and severity. It is important to appreciate that flow reversal is occasionally seen in the setting of rapid elastic recoil of the descending Ao and/or aortic stiffness of the abdominal Ao even in the absence of important AR [28, 29].

**Table 7:**
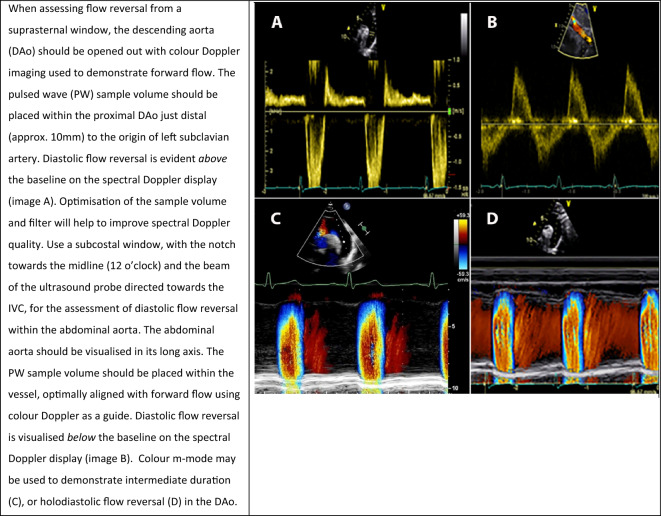
Assessment of flow reversal in the descending thoracic aorta and abdominal aorta

### Semi-quantitative parameters

#### Vena contracta width

**Table:** 

**Key points**
• The vena contracta (VC) width represents the narrowest portion of the regurgitant jet and is a surrogate for the effective regurgitant orifice area
• A VC < 0.3cm is consistent with mild or less AR, and a VC > 0.6cm is consistent with severe AR
• VC width may under- or over-estimate AR severity in the context of a non-circular regurgitant orifice
• Three dimensional (3D) VC may be considered when imaging windows allow, particularly if undertaking transoesophageal echocardiography

**Fig. 3:**
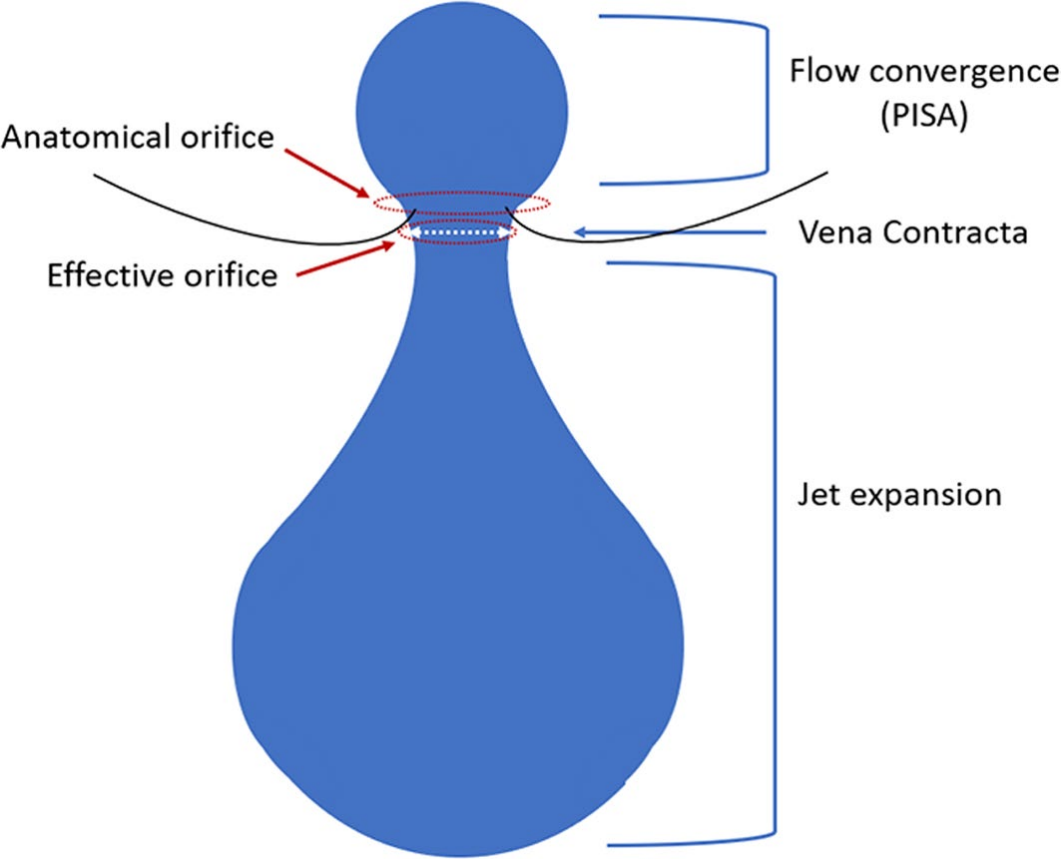
Demonstrates the three constituent parts: flow convergence, the vena contracta, and jet expansion (blue arrows). The true anatomical regurgitant orifice compared with the effective regurgitant orifice is identified (red arrows)

Regurgitant jets have three constituent parts: the flow convergence zone, the vena contracta (VC), and jet expansion (Fig. 3). The VC represents the narrowest portion or the ‘neck’ of the jet, and it is directly related to the size of the regurgitant orifice. It is important to appreciate that the VC **does not** occur at the level of the cusp tips, but immediately downstream: when blood flows through an orifice, the jet continues to ‘contract’ for a short duration. The *anatomical orifice* is what allows regurgitation to occur, and whilst the *effective orifice* is smaller (as the jet initially contracts prior to expansion), it is the *effective orifice* or *effective regurgitant orifice area* (EROA) that correlates with AR severity and prognosis (Fig. 3) [30]. VC width provides a surrogate measure for the EROA when the effective orifice is circular, and the BSE recommends that, wherever possible, an assessment of VC should be made in all patients with more than mild AR.

The methodology for obtaining and optimising VC width is demonstrated in Table 8. An important limitation of this measure is that it assumes that the regurgitant orifice is circular, whereas in practice this is frequently not the case. As such, assessing the VC width from a single 2D window may lead to either under- or over-estimation of the EROA. Use of 3D echo has the potential to overcome this limitation, but the temporal resolution and low frame rates of 3D colour flow Doppler imaging may reduce the accuracy of the measurement. Nonetheless, using multi-beat acquisitions during a breath hold and narrowing the acquisition volume to include only the VC can allow quantifiable 3D vena contracta areas (VCA) [31–33]. Recent studies suggest that the cutoff for 3D VCA that best correlates with severe AR is ~ 0.30 mm^2^ [31–33]. Although the BSE currently **does not** recommend routine use of 3D VCA, there is growing evidence of its clinical utility, particularly when using transoesophageal echocardiography.

**Table 8:**
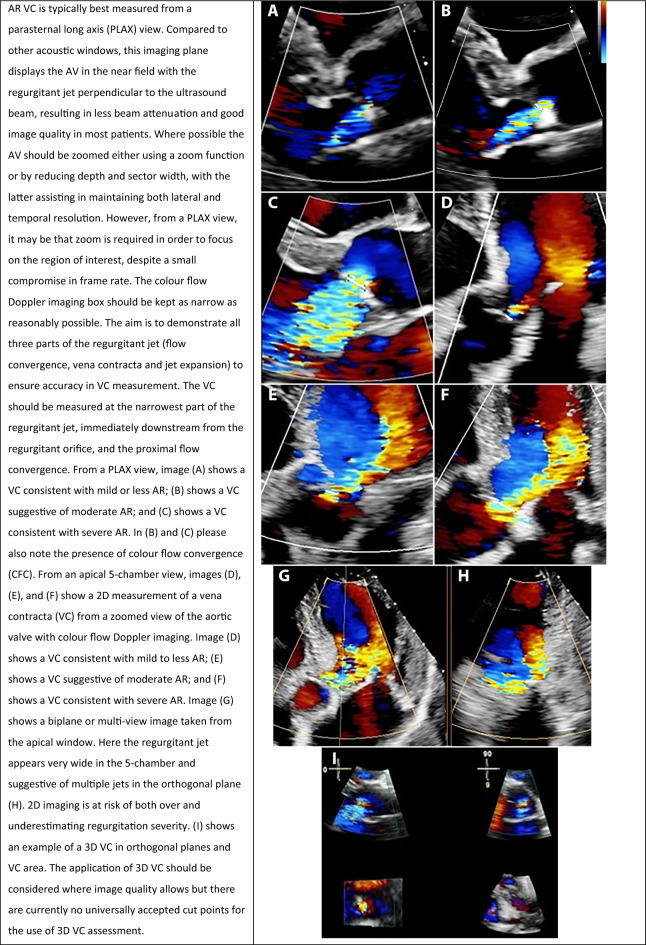
Vena contracta (VC) width: how best to approach this parameter in the assessment of aortic regurgitation (AR) severity

#### Pressure half time

**Table:** 

**Key points**
• Pressure half time (PHT) represents the time taken for the aortic-left ventricular (LV) pressure difference to fall to half its initial value
• A PHT of < 200ms is consistent with severe aortic regurgitation (AR)
• A PHTof > 500ms is consistent with mild or less AR
• LV compliance, LV dysfunction and hypertensive therapies may all affect the accuracy of the calculated PHT

Pressure half time (PHT) is assessed using continuous wave (CW) Doppler and represents the time taken for aortic-left ventricular (LV) pressure difference to fall to half its initial value. The more severe the AR, the faster the equalisation of pressure, the steeper the AR deceleration slope and the shorter the PHT (Table 9). An accurate assessment of PHT requires a complete CW envelope; as such, a poorly aligned Doppler cursor, eccentric jets and multiple jets will impact on the accuracy of this measurement. A typical pressure difference between the Ao and LV will lead to an early diastolic peak velocity of approximately 4m/s on CW Doppler. If the early diastolic peak velocity is lower, this may suggest poor alignment between the regurgitant lesion and the CW Doppler cursor, deeming the measurement of PHT inaccurate (Table 9, (B)).

**Table 9:**
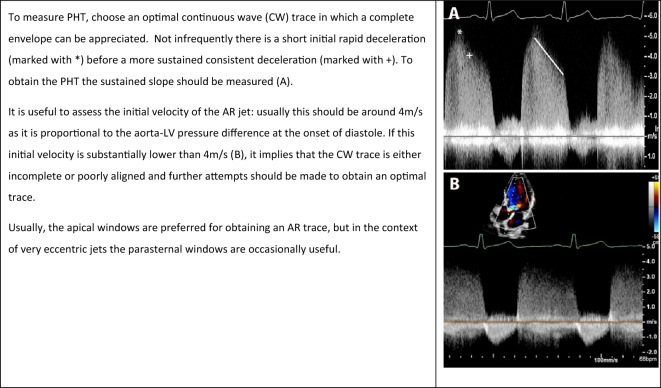
Considerations when using pressure half time (PHT) to determine aortic regurgitation severity

Another important limitation is that when LV diastolic filling pressure is increased, the pressure difference between the Ao and LV is reduced, resulting in a short PHT even in the absence of severe AR [34]. Other important haemodynamic factors which may influence PHT include stroke volume (SV), compliance of the Ao, and LV performance [34]. Hypertensive therapies such as vasodilators can also reduce the Ao-LV gradient by lowering diastolic pressure independent of AR. This guideline recommends that PHT be used only as part of a multi-parametric assessment of AR severity.

### Quantitative parameters

#### Effective regurgitant orifice area

**Table:** 

**Key points**
• An effective regurgitant orifice area (EROA) of > 0.3 cm^2^ is consistent with severe AR
• Accurate calculation of EROA using the PISA method is limited in the setting of multiple and eccentric jets

The effective regurgitant orifice area (EROA) is a direct estimation of the size of the regurgitant orifice. This is a quantitative tool which is clearly linked with prognosis in chronic severe AR [35]. This guideline recommends that an attempt is made to calculate EROA in all patients with more than mild AR.

There are two methods by which the EROA can be assessed. The first is a more direct technique using the proximal isovelocity surface area (PISA) method, which should be considered the default approach. The second indirect method is described in the ‘Regurgitant volume’ section (below) and is subject to greater error.

The PISA method requires both colour flow Doppler and spectral Doppler imaging (Table 10). The radius of the CFC hemisphere is measured to calculate the CFC surface area and is then multiplied by the Nyquist limit, providing an estimate of the *AR flow rate*. The Nyquist limit baseline should be moved in the direction of regurgitant flow to between 0.2 and 0.4 cm/s; this optimises the hemisphere definition and facilitates measurement accuracy (Table 10 (B)). This is **not** to be confused with reducing the colour scale which will reduce the Nyquist limit but will not lead to a clearer hemisphere of isovelocity flow. The CFC hemisphere radius should be measured in the direction of the ultrasound beam, and **not** in the direction of the regurgitant jet [36]. Using CW Doppler, an AR envelope should be obtained. By tracing the regurgitant envelope it is possible to obtain both the velocity time integral (VTI) and the *AR peak velocity*. The *AR flow rate* is then divided by the *AR peak velocity* to calculate EROA (Box 1).

**Box 1:** The calculation used to calculate the effective regurgitant orifice area (EROA) using the proximal isovelocity surface area (PISA) method.

$${\mathbf{EROA \quad = }} \quad \left( {{\mathbf{2 } }{{\varvec\pi}} {\mathbf{ r}}^{\mathbf{2}} {\mathbf{x V}}_{{\mathbf{N}}} } \right)/{\mathbf{V}}_{{{\mathbf{AR}}}}$$
Where r = radius, V_N_ = Nyquist velocity, V_AR_ = peak early diastolic velocity of the AR CW jet.

**Table 10:**
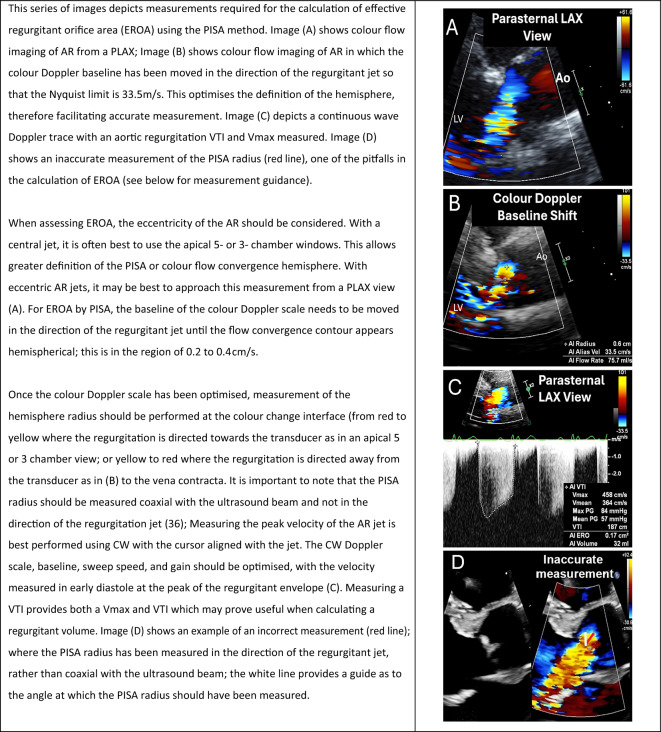
Effective regurgitant orifice area: how to approach measurements

There are several limitations with this technique: firstly, in the context of multiple AR jets, the largest jet should be identified and used for assessment, although this will likely underestimate overall severity. With eccentric jets, small errors in radius measurements are multiplied and may result in large inaccuracies in the calculation of EROA. The presence of calcification, and a non-circular regurgitant orifice (commonly seen in BAVs) with a hemi-elliptic PISA will all impact accuracy.

#### Regurgitant volume

**Table:** 

**Key points**
• This guideline recommends calculation of regurgitant volume (RVol) using the PISA methodology where an accurate measurement of the PISA radius and regurgitant jet VTI can be obtained
• A RVol of < 30mL is consistent with mild AR
• A RVol of > 60mL is consistent with severe AR

Regurgitant volume (RVol) is a useful quantitative parameter providing prognostic information in AR. Along with EROA, it is a quantitative tool that provides prognostic information. There are two ways to estimate the RVol; the PISA method is the preferred approach, whereas the continuity method is more challenging and subject to greater error. Both techniques require a high degree of skill and excellent image quality and therefore an accurate assessment may not be possible.

**Fig. 4:**
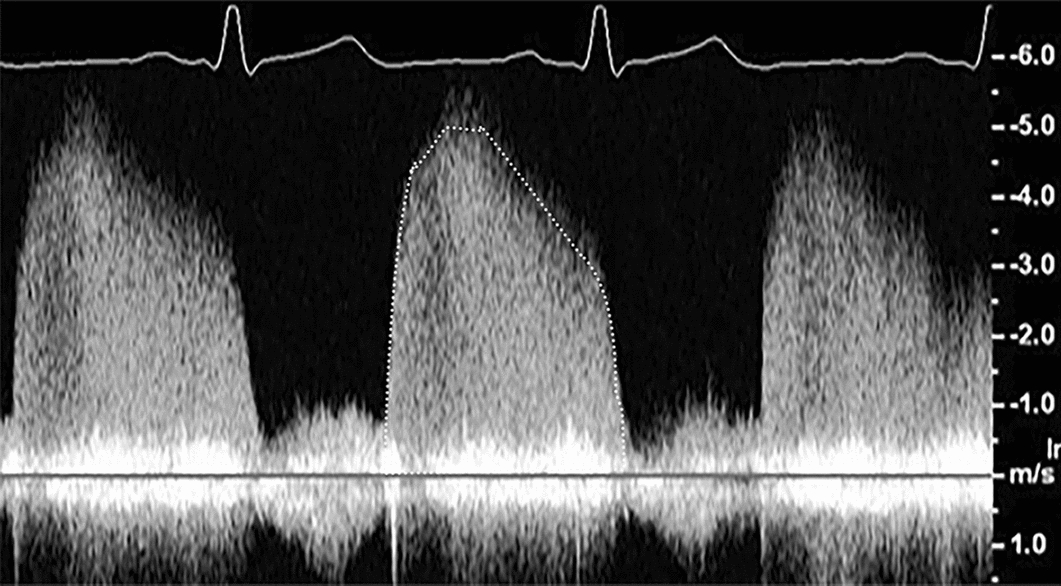
This image shows continuous wave Doppler of the aortic regurgitation trace. The velocity time integral (VTI) of the regurgitant waveform has been traced. The calculated VTI multiplied by the effective regurgitant orifice area (calculated as shown in Table 10) estimates the regurgitant volume. Of note, on some ultrasound systems, measurement of the VTI will also provide an estimate of the peak early diastolic velocity of the AR jet

To calculate the RVol using the PISA method, the EROA should be derived as described previously. In addition, the VTI of the regurgitant waveform should be obtained (Fig. 4). The RVol can then be calculated as the product of EROA and VTI_AR_ (Box 2).

**Box 2:** Outlines the quantitative PISA method used to calculate regurgitant volume (RVol).

$${\mathbf{RVol}} = {\mathbf{EROA x VTI}}_{{{\mathbf{AR}}}} \quad {\mathbf{where \; EROA}} = \left( {{\mathbf{2} }{\varvec\pi} {\mathbf{ r}}^{\mathbf{2}} {\mathbf{x V}}_{{\mathbf{N}}} } \right)/{\mathbf{V}}_{{{\mathbf{AR}}}}$$
where EROA = effective regurgitant orifice area, VTI_AR_ = velocity time integral of the regurgitant jet, r = radius, V_N_ = Nyquist velocity, V_AR_ = peak early diastolic velocity of the AR CW jet.

The second method uses the continuity principle (Table 11). In normal circumstances, the SV through the mitral valve (SV_MVA_) is the same as the SV through the AV (SV_AV_). If there is a sufficiently large volume of AR, there will be an isolated increase in SV_AV_. Using the continuity principle, the aortic regurgitant volume can then be derived as SV_AV_-SV_MVA_. The continuity method **cannot** be used where there is significant concomitant mitral regurgitation (MR). Additionally, this method should not be used where there is interference of the Doppler trace, secondary to the AR (i.e. very severe posteriorly directed AR causes restriction of the MV opening (pseudo functional mitral stenosis), thus invalidating the use of the PW at the MV annulus). In such a scenario, right ventricular outflow tract (RVOT) SV could be used instead of SV_MVA_. The application of the continuity equation brings with it additional limitations related to the uniformity of flow profiles, the accuracy of sample volume placement and assumptions in relation to orifice shape. If the RVol is obtained using the continuity method, the EROA can then be derived as in Box 3.

**Box 3:** Outlines the calculation used to calculate regurgitant volume (RVol) using the continuity principle.

$${\mathbf{RVol }} = {\mathbf{SV}}_{{{\mathbf{Ao}}}} {-}{\mathbf{ SV}}_{{{\mathbf{MVA}} }} \quad {\mathbf{where SV}}_{{{\mathbf{Ao}}}} = {\mathbf{CSA}}_{{{\mathbf{LVOT}}}} \times {\mathbf{VTI}}_{{{\mathbf{LVOT}}}}$$
where SV_Ao_ = the stroke volume through the aorta; SV_MVA_ = stroke volume through the mitral valve annulus; CSA = cross sectional area; VTI = velocity time integral.

**Table 11:**
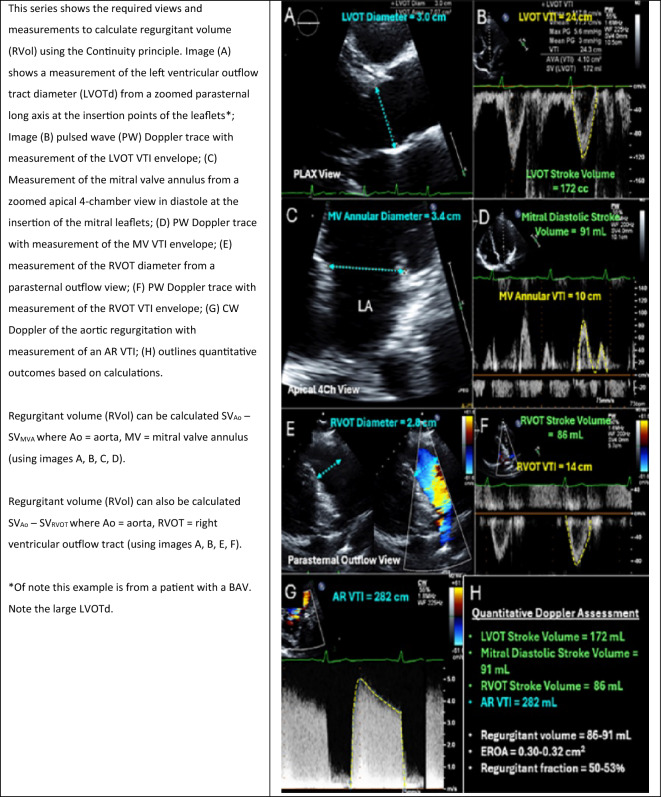
Calculating the regurgitant volume using the continuity principle

#### Regurgitant fraction

**Table:** 

**Key points**
• Regurgitant fraction (RF) is the proportion of regurgitant volume compared to the forward stroke volume expressed as a percentage
• A RF of < 30% is consistent with mild aortic regurgitation
• A RF of > 50% is consistent with severe aortic regurgitation

Regurgitant fraction (RF) is the ratio of the RVol to the forward SV expressed as a percentage (see Box 4). RVol and SV can be estimated using the approaches outlined above. There is some evidence that RF is a useful assessment of AR severity in the context of impaired LV systolic function or reduced cardiac output, when even a relatively small RVol is proportionally more significant owing to the reduced forward SV [6].

**Box 4:** Outlines the calculation used to calculate regurgitant fraction (RF).

$${\mathbf{RF}} = {\mathbf{RVol}}/{\mathbf{SV}} \times {\mathbf{100}}$$
where RVol = regurgitant volume, SV = stroke volume.

## Additional key parameters in the assessment of chronic aortic regurgitation

**Table:** 

**Key points**
• Severe chronic aortic regurgitation is **almost always** associated with left ventricular (LV) dilatation
• All patients should have an assessment of both indexed LV volumes and indexed LV linear dimensions
• All patients should have a comprehensive assessment of the aorta including indexed aortic dimensions
• Global longitudinal strain (GLS) may enable the identification of early LV dysfunction

### LV size and function

In the context of chronic AR, the left ventricle (LV) is exposed to increased preload, leading to adaptive changes including progressive dilatation and eccentric left ventricular hypertrophy (LVH). A normal indexed LV volume in and of itself therefore **almost always** rules out severe AR, except in rare circumstances including acute AR, which is addressed in a later section [37].

As AR progresses, LV dilatation eventually becomes maladaptive, at which point LV function will become impaired, patients may develop symptoms, and prognosis is compromised. As such, accurate quantification of LV size and function is essential, and it is recommended that, wherever possible, LV ejection fraction (LVEF) and indexed LV volumes should be obtained and reported using the Simpson’s biplane method for all patients.

The BSE additionally recommends that both non-indexed *and* indexed LV linear dimensions are reported. There is a wealth of data supporting the use of these parameters in the management of AR, and they should be included on the echocardiographic report to support patient management [6, 38–41].

### Global longitudinal strain (GLS)

Global longitudinal strain (GLS) obtained using speckle tissue tracking may provide an indication of early LV dysfunction [42–44]. As with other forms of heart valve disease, GLS in patients with AR becomes reduced as the disease progresses [45, 46]. Whilst lower maximal values of GLS are associated with poorer outcomes in chronic severe AR, as yet there is no accepted threshold of GLS that should be used to prompt a change in clinical management. However, in patients undergoing serial assessment, it is reasonable to highlight progressive reductions in GLS as this may prompt closer clinical or echocardiographic surveillance.

### Aortic dimensions

A comprehensive assessment of the aorta (Ao) is essential in the context of AR. Firstly, because aortic dilatation may itself be the *cause* of the regurgitation (Type I AR; see Sect. 2 ‘Mechanism of aortic regurgitation’); secondly, variant anatomy such as BAV is associated with aortic enlargement; and finally, the presence and pattern of aortic dilatation radically alters the potential approach to aortic valve intervention. Whilst transthoracic echocardiography (TTE) provides an excellent standard of quality in the evaluation of aortic dimensions, it is important to appreciate the limitations of this modality when it comes to the assessment of the Ao as a whole, with the proximal descending thoracic Ao in particular extremely challenging to visualise with TTE alone. As previously recommended by the BSE, the aortic root (AoR) and ascending aorta (AscAo) should be measured from the PLAX window at three levels: the Sinus of Valsalva (SoV); the sino-tubular junction (STJ); and the AsAo defined as a level 1cm above the STJ (Table 12) [9, 47]. For optimal visualisation it may be that a higher anatomical window is required. Measurements should be performed at end-diastole, defined as the onset of the QRS on the surface electrocardiogram (ECG), using an inner-edge to inner-edge approach, with the measurement taken perpendicular to the major axis of the aorta. The BSE recommends that both absolute values and indexed aortic dimensions scaled to height are reported [47]. The aorta shape and contours as well as the absence or presence of an aortic dissection flap should be described within reports, with appropriate clinical escalation.

**Table 12:**
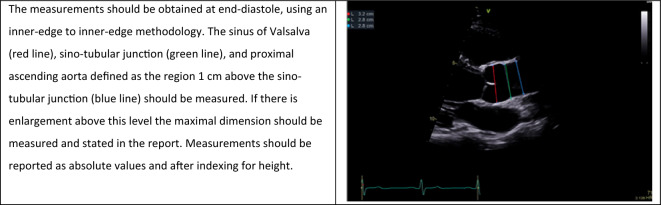
Assessment of aortic root dimensions. [47] Reproduced with permission from Harkness et al.

## Echocardiographic features that may prompt referral for intervention

The timing of intervention for severe aortic regurgitation (AR) is a clinical decision and therefore beyond the remit of this guideline. The commonest indication for surgery is the presence of cardiovascular symptoms, in which case a comprehensive echo with accurate assessment of AR severity, LV function, concomitant valve disease and an assessment of the likelihood of pulmonary hypertension is essential to aid surgical planning.

In asymptomatic severe AR, several echocardiographic findings play a crucial role in determining surgical eligibility, including indexed LV linear dimensions, LV ejection fraction (EF), and aortic (Ao) dimensions. It is useful for echocardiographers to be aware of these specific high-risk criteria and highlight them in the conclusion of the comprehensive echo report, thereby supporting optimal patient management.

For those patients with asymptomatic severe AR, it is vital to ensure any changes in LV size or function are escalated as these findings are predictive of the development of heart failure, and significant determinants of survival and functional outcome following surgical intervention [22].

In terms of aortic dilatation, the valve morphology (i.e. bicuspid vs. tricuspid valves) and the presence of an underlying connective tissue or genetic disorder all play a role in determining the threshold for surgical intervention. An Ao or ascending aorta (AscAo) dimension of ≥ 55mm in all patients is an indication for surgery. Special populations such as patients with Marfan syndrome, a BAV or aortic coarctation have a lower threshold, ranging between 45-50mm (and rarely 40mm) [48]. An aortic dimension ≥ 45mm in the presence of severe AR would usually prompt concomitant AoR replacement at the time of aortic valve intervention. Table 13 provides an overview of these high-risk echocardiographic criteria.

**Table 13:** High risk echocardiographic features that may prompt referral for intervention

Red flags
LVESd > 50 mm
LVESdi ≥ 25 mm/m^2^
LVEF ≤ 55%
Ascending aorta diameter ≥ 55 mm in all patients
Ascending aorta diameter ≥ 45 mm in special populations
Rapid dilatation of the LV approaching surgical threshold

*LVESd* left ventricular end systolic dimension, *LVESdi* left ventricular end systolic dimension indexed, *LVEF* left ventricular ejection fraction, *LV* left ventricle

## Approach to the patient with chronic AR

**Table:** 

**Key points**	
• Aortic regurgitation (AR) should be graded as mild, moderate, or severe	
• The grading of AR severity should be determined using a multiparametric approach as outlined in Fig. 5	
• AR tends to progress slowly, but interval echocardiographic surveillance is advised, in particular for moderate and severe AR

**Fig. 5:**
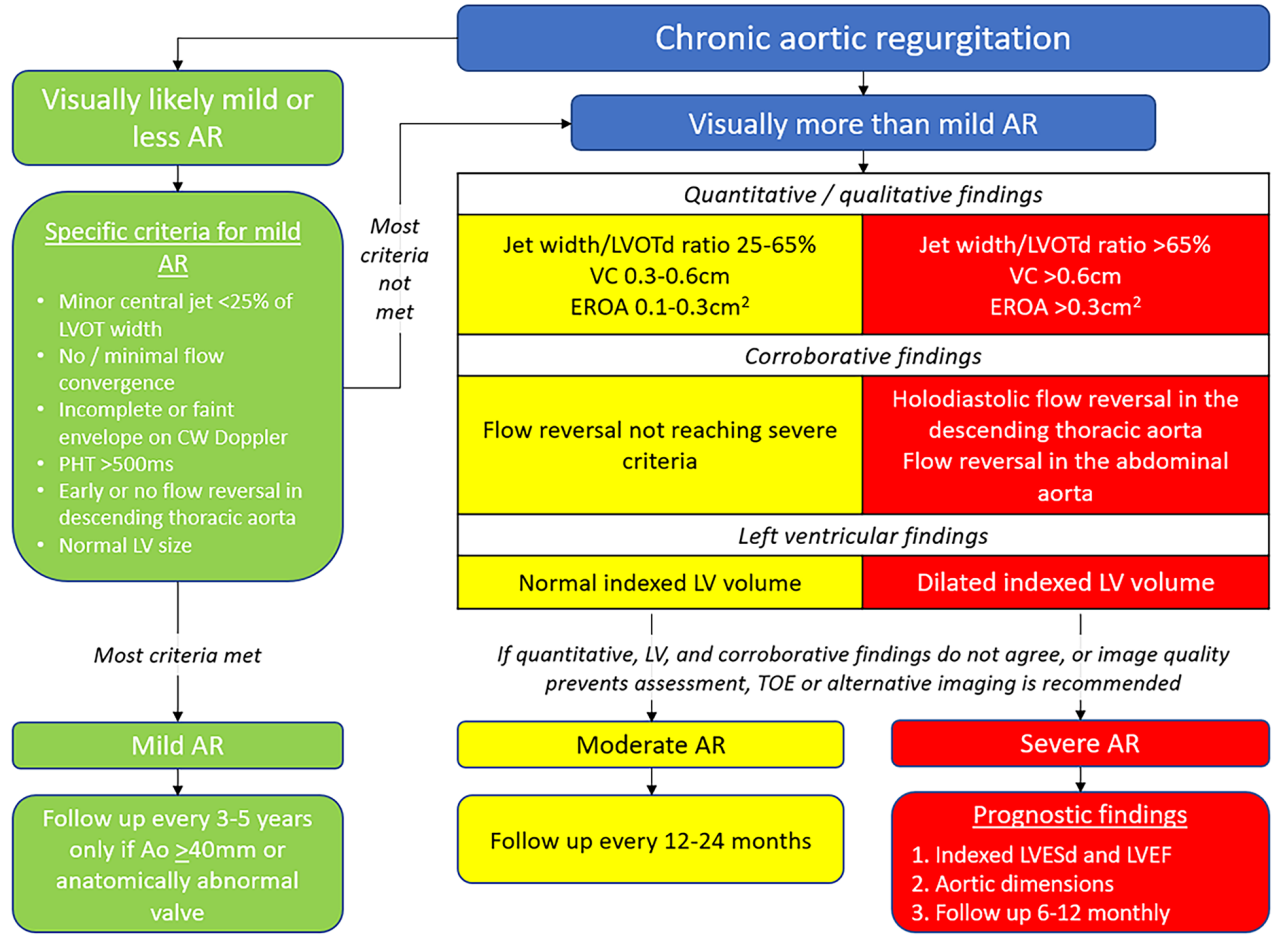
Decision aid to guide assessment of aortic regurgitation severity. If AR visually appears mild or less in severity, this should be clarified using a selection of the specific criteria as listed in the green box. If confirmed, there is no need to attempt further assessment. In those patients where AR appears more than mild, further detailed evaluation is required. In this scenario, the severity of AR can be confirmed if at least one *quantitative* / *qualitative* characteristic, one *corroborative* characteristic, and *indexed left ventricular volumes* are all in agreement. For example: a patient with a VC of 0.7cm, holodiastolic flow reversal in the descending thoracic aorta, and dilated indexed LV volumes, clearly has severe AR. In this situation the focus should then be on key prognostic features including the indexed LVESd, LV function, and aortic dimensions. If quantitative/qualitative characteristics, corroborative characteristics, and indexed LV volumes are *not* in agreement, further imaging such as TOE is recommended to clarify severity and ensure optimal management of the patient. In a significant minority of cases, quantitative assessment will not be possible, either because image quality is insufficient, or other factors such as valve calcification impedes detailed quantification. In such cases, the BSE recommend that further imaging (such as TOE) is advised within the report. This ensures appropriate classification of AR severity, optimal timing of follow-up and promotes good clinical management. (AR = aortic regurgitation; LVOT = left ventricular outflow tract; Ao = aorta; CW = continuous wave; PHT = pressure half time; LV = left ventricular; LVOTd = left ventricular outflow tract diameter; VC = vena contract; EROA = effective regurgitation office area; LVESd = left ventricular end systolic dimension; LVEF = left ventricular ejection fraction; TOE = transoesophageal echocardiography)

This guideline recommends that aortic regurgitation (AR) is graded as either mild, moderate, or severe. Table 14 summarises the severity thresholds for the echocardiographic parameters of AR. The progression from mild to moderate to severe AR tends to occur slowly, although the rate of progression may vary according to the mechanism and underlying aetiology of valve insufficiency. This guideline recommends that all patients with moderate or severe AR undergo both clinical and echocardiographic surveillance: those with moderate AR should be seen every 12–24 months; severe AR not meeting surgical thresholds every 6–12 months [49]. Where the degree of AR is mild in the setting either an anatomically abnormal AV or an AoR ≥ 40mm, follow up should be at 3–5 years [50]. Patients with an anatomically normal AV and a AoR < 40mm do not require routine follow up [50]. Figure 5 outlines and summarises the echocardiographic approach when faced with a patient with chronic AR.

**Table 14:** Parameters by method and severity grading recommended by the British Society of Echocardiography

Method	Mild	Moderate	Severe
Continuous wave (CW) jet density	Incomplete or faint	Dense	Dense
Colour flow convergence (CFC)	None/very small		Large
Pressure half time (PHT) (ms)	> 500	200–500	< 200
Jet width/LVOTd (%)	< 25	25–65	> 65
Diastolic flow reversal in the DAo (cm/s)	Brief / none	Intermediate	Prominent holodiastolic ≥ 20cm/s velocity
Vena contract width (cm)	< 0.3	0.3–0.6	> 0.6
Effective regurgitant orifice area (EROA) (cm^2^)	< 0.10	0.10–0.30	> 0.30
Regurgitant volume (RVol) (mL)	< 30	30–60	> 60
Regurgitant fraction (RF) (%)	< 30	30–50	> 50
Left ventricular (LV) size	Normal		Dilated

## Additional cardiac imaging

**Table:** 

**Key points**
• TOE can be used to complement TTE, providing clarity in relation to valve morphology and mechanism where image quality is suboptimal, or when surgical AV intervention is complex
• CMR is less susceptible to the acoustic window limitations of TTE and can provide useful information in relation to LV volumes, regurgitant volumes and the quantification of cardiac remodelling
• Cardiac CT is useful in the evaluation of aortic size and shape

### Transoesophageal echocardiography (TOE)

In many instances, TTE is sufficient to perform a comprehensive assessment of AR. Where image quality does not allow, or in circumstances where the *quantitative / qualitative, corroborative, and LV volume* do not align with regards to AR severity (see Fig. 5), additional imaging is recommended. TOE is a useful adjunct to help clarify valve morphology, regurgitation mechanism and severity, and provides assessment for possible infection or dissection. TOE should also be considered pre-surgery where patients are undergoing aortic valve sparing or repair surgery, or any percutaneous procedures. TOE should include both 2D and 3D imaging as well as colour and spectral Doppler modalities. The BSE minimum dataset for a standard transoesophageal echocardiogram should be performed with the AR specific echocardiographic images outlined in Table 15 [51].

**Table 15:**
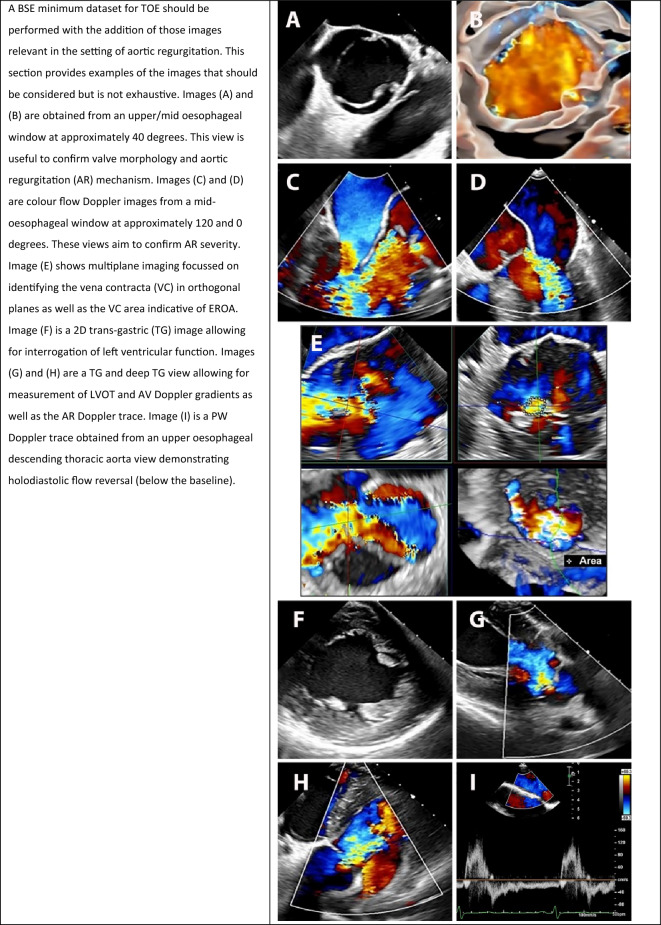
Transoesophageal echocardiographic (TOE) images which complement transthoracic echocardiography

### Cardiac magnetic resonance (CMR) imaging

Although echocardiography is the first-line diagnostic imaging modality for patients with AR, cardiac magnetic resonance imaging (CMR) can provide additional useful information. Particularly where TTE quality is poor, CMR can provide information to clarify discordant or discrepant data. CMR, which offers greater inter-study reproducibility in relation to LV volumes, can also evaluate regurgitant volumes and AR severity, the underlying regurgitation mechanism, SV, EF, and the quantification of cardiac remodelling [3, 52]. CMR also provides accurate data in the assessment of aortic dimensions.

### Cardiac computed tomography (CT)

Cardiac computed tomography (CT) provides fast, high-resolution images of the aortic root and thoracic aorta allowing for accurate and reproducible assessment of size and shape, and is considered the modality of choice for this anatomical structure [53]. Cardiac CT can also be used to determine AV morphology, locate and quantify calcification, and identify the presence of an aortic coarctation or dissection [3].

#### Exercise stress echocardiography

**Table:** 

**Key points**
• Exercise stress echocardiography (ESE) should **not be used** to evaluate aortic regurgitation severity
• ESE may be useful for assessment of contractile reserve
• ESE may be useful for unmasking cardiovascular symptoms or alternative pathologies

Exercise stress echocardiography (ESE) is **not useful** in the evaluation of severity of AR [54, 55]. During exercise, heart rate increases which reduces the duration of diastole. Consequently at peak stress, AR is less obvious and AR severity may be underestimated [56]. However, ESE may be useful for the assessment of contractile reserve (CR) [54, 57]. In asymptomatic patients with severe AR, a lack of CR predicts adverse outcomes [57–59]. ESE also provides opportunities for the unmasking of symptoms, precipitation of exercise-induced arrythmias, and overall functional capacity [54], or may identify other causes for symptoms such as ischaemia [54]. Where there is a discrepancy between symptoms and AR severity, ESE may assist in clarifying this discrepancy.

## Acute aortic regurgitation

**Table:** 

**Key points**
• The assessment of acute severe aortic regurgitation (AR) is very different from chronic AR
• Patients with acute severe AR are often *in extremis* and imaging is often challenging
• The left ventricle (LV) is often normal sized with normal or hyperdynamic LV systolic function

Acute aortic regurgitation (AR) differs from chronic AR in several important ways. The aetiology is often very different: infective endocarditis and aortic dissection are the most frequent causes of acute severe AR; less often it occurs as a complication of transcatheter procedures or blunt chest trauma [60, 61]. Because chronic AR progresses over many years, there is sufficient time for compensatory LV changes to develop. Acute severe AR allows no such compensation; consequently, LV filling pressures and left atrial pressures become significantly elevated. Patients with acute severe AR usually present *in extremis* and frequently will be in overt or incipient cardiogenic shock, pulmonary oedema, or both [62]. As such, they are often cared for in a high intensity setting such as intensive or coronary care, they may be receiving circulatory or respiratory support, and obtaining high-quality echo images will be particularly challenging. A high index of suspicion is required when undertaking echocardiography in such patients to ensure that acute severe AR is not missed.

**Fig. 6:**
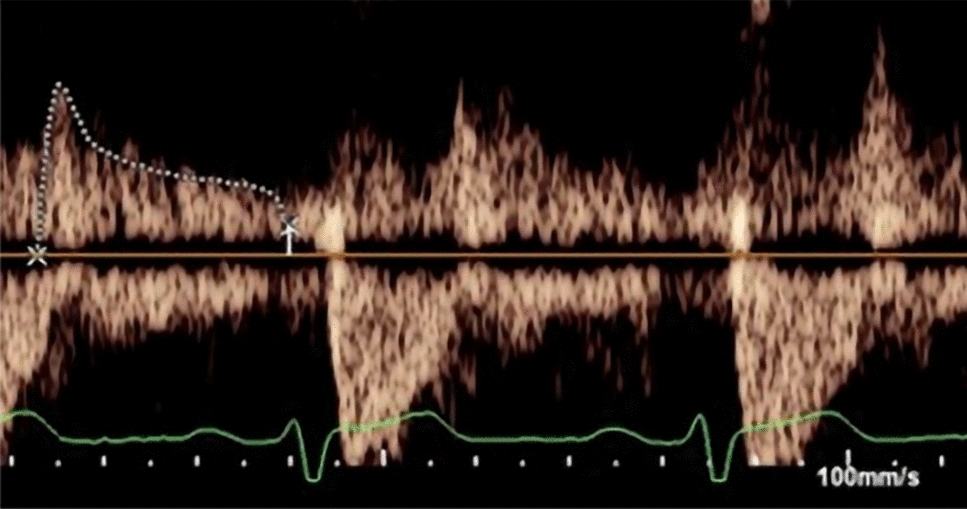
A velocity time integral (VTI) envelope of the diastolic flow reversal from the descending thoracic aorta. Drawing a comparison between the reversal and the forward stroke volume VTI from the descending aorta provides a semi-quantitative approach to determining regurgitant fraction (RF)

Echocardiographically, the LV will **not** necessarily be dilated, and often there will be normal or even hyperdynamic systolic function (despite the presence of pulmonary oedema) with concordant increases in LVOT velocities and the LVOT VTI. Additionally, there may be premature closure of the AV and premature termination of aortic flow as well as more indirect signs of acute severe AR including decreased transmitral deceleration time, premature closure of the MV, and diastolic MR with the latter shown to be a predictor of decompensation (Table 16) [61, 63]. A useful semi-quantitative estimate of regurgitation fraction can also be established by comparing the forward stroke volume with the degree of holodiastolic flow reversal in the descending thoracic aorta (DAo) (Fig. 6). However, in the setting of very severe acute AR, due to markedly elevated left ventricular end diastolic pressures (LVEDP) and equalisation of LV and aortic end diastolic pressures, it is possible that there is no pressure difference thus resulting in curtailing of any diastolic flow reversal in the DAo. In acute severe AR, in light of the LVEDP rising rapidly, SV cannot be maintained despite intrinsic compensatory mechanisms [64]. Echocardiographic findings consistent with acute severe AR need to be escalated urgently.

**Table 16:** Classical features of aortic regurgitation in the chronic and acute setting

Chronic AR	Acute AR
Increasing LV size (LVEDd, LVEDV, LVESd, LVESV)	LV size is not dilated
LV remodeling (shape becomes more spherical)	Hyperdynamic LV systolic function
Deteriorating LV systolic function and LVEF	Increased LVOT VTI / Vmax
Increased wall mass	Premature closure of the MV
Normal LVEDP	Diastolic MR
	High LVEDP
	Decreased transmitral deceleration time
	Premature termination of diastolic flow (AR duration may be brief)

*LV* left ventricular, *LVEDd* left ventricular end diastolic dimension, *LVEDV* left ventricular end diastolic volume, *LVESd* left ventricular end systolic dimension, *LVESV* left ventricular end systolic volume, *LVEF* left ventricular ejection fraction, *LVEDP* left ventricular end diastolic pressure, *LVOT VTI* left ventricular outflow tract velocity time integral, *Vmax* velocity maximum, *MV* mitral valve, *MR* mitral regurgitation, *AR* aortic regurgitation

## Combined valve disease

**Table:** 

**Key points**
• In the setting of combined valve disease, a comprehensive assessment of all valve pathologies is mandatory
• The severity and prognosis of mixed aortic valve disease is closely related to the AV Vmax
• Any high-risk feature of **either aortic stenosis or aortic regurgitation** should prompt intervention for mixed aortic valve disease
• Concomitant AR/MR frequently leads to LV dysfunction so careful monitoring of left ventricular ejection fraction (LVEF) for even subtle deterioration is recommended

### Aortic regurgitation and aortic stenosis

AR combined with aortic stenosis (AS) is often referred to as mixed aortic valve disease (MAVD). MAVD is common due to the increased prevalence of AS within the aging population and other known aetiologies which cause dual pathologies. MAVD can lead to challenges in determining disease severity. Protocols typically provide guidance in the assessment of pathologies in isolation, but it is important to question where moderate AS and moderate AR coexist should the total valve lesion be considered severe, given the prognosis of this combination of valve lesions is more in keeping with isolated severe AS [65, 66]. In the context of mixed AV disease (defined as the presence of at least moderate AR and AS), the key parameter that predicts time to event is AV Vmax [65, 67]. Valve area, and indices of AR severity appear less well associated with outcomes. In mixed AV disease, the lesion should be reported as ‘moderate / severe / very severe mixed AV disease’ according to the AV Vmax (3/4/5 m/s respectively) [65, 67]. AV Vmax needs to be considered in the setting of adverse LV remodelling and declining EF, with this high risk group demonstrating a higher incidence of death and heart failure admissions [68]. Regardless of the presence of MAVD, current recommendations still suggest that conventional recommendations for AS and AR be used to guide surgical intervention [65, 67].

### Aortic regurgitation and mitral regurgitation

Combined AR and mitral regurgitation (MR) is also common given the prevalence of MR. Previously there has been concern that combined AR/MR may result in myocardial structural changes, pulmonary hypertension, or arrhythmias prior to the development of symptoms and guideline driven eligibility for surgery [69]. Moreover, there was anxiety that the development of these endpoints may be irreversible. Concomitant AR/MR is an example of a common but understudied multiple valve interaction. There is limited data to guide management but due to both pressure and volume overload, LV dysfunction is frequent [70]. The BSE recommend a comprehensive assessment of both valves (as outlined by corresponding guidelines) and evaluation of LV size and systolic function, and SV [71]. Careful monitoring of LV function is crucial and even subtle deterioration in LVEF may suggest consideration of surgical intervention. There is data to suggest that LV dilation due to chronic AR may not be fully reversible and as such where AV intervention is deemed suitable, it would be prudent to consider MV intervention as required [72].

## Suggested reporting template

Aside from the acquisition of accurate echocardiography images, it is essential to ensure that echo findings are communicated via a comprehensive, concise and relevant report. Reports should include comments on all cardiac structures interrogated. Where AR is present, it is crucial to ensure the key parameters are highlighted (Table 17).

**Table 17:** Suggested reporting template for aortic regurgitation

Section	Data / detail
Demographics	Height, weight, body surface area (BSA)
	Blood pressure, heart rate and rhythm
Image quality	Good, fair, poor
Aortic valve morphology	Tricuspid / bicuspid (± raphe) / uni or quadricuspid
	Leaflet abnormalities (restriction / prolapse / calcification / perforation, or wide coaptation defect)
Aortic root	Size, indexed/scaled size, shape, number of sinuses
Aortic regurgitation severity	Jet width / LVOT diameter ratio
	Vena contracta width
	Pressure half time
	Regurgitant volume and/or regurgitant fraction
	Effective regurgitant orifice area
	Presence of flow convergence colour Doppler
	Continuous wave jet density
	Presence and severity of diastolic flow reversal in the descending aorta and abdominal aorta
Additional prognostic markers	Left ventricular dimensions and volumes as absolute and indexed values Left ventricular systolic function and ejection fraction (EF)
	Global longitudinal strain where available
Aortic stenosis	Note presence and severity (see specific BSE guidance) [13]

*LVOT* left ventricular outflow tract, *EF* ejection fraction, *BSE* British Society of Echocardiography

## Conclusion

The prevalence of AR within the population is increasing. As such the role of transthoracic echocardiography in the identification and assessment of AR becomes increasingly important. High quality, accurate, and reproducible echocardiographic images, measurements, and calculations will facilitate improved determination of AR severity. This will ultimately improve decision making in determining the optimal time for intervention, resulting in improved outcomes for patients [6, 73]. 

## Data Availability

No datasets were generated or analysed during the current study.

## Abbreviations

2D: Two dimensional
3D: Three dimensional
A2C: Apical two chamber
A3C: Apical three chamber
A4C: Apical four chamber
A5C: Apical five chamber
Ao: Aorta
AoR: Aortic root
AR: Aortic regurgitation
AS: Aortic stenosis
AscAo: Ascending aorta
AV: Aortic valve
BAV: Bicuspid aortic valve
BP: Blood pressure
BSA: Body surface area
BSE: British Society of Echocardiography
CFC: Colour flow convergence
CMR: Cardiac magnetic resonance imaging
CR: Contractile reserve
CT: Computed tomography
CW: Continuous wave Doppler
DAo: Descending thoracic aorta
EF: Ejection fraction
ESE: Exercise stress echocardiography
GLS: Global longitudinal strain
LV: Left ventricle
LVEDd: Left ventricular end diastolic dimension
LVEDdi: Left ventricular end diastolic dimension indexed
LVEDP: Left ventricular end diastolic pressure
LVEDV: Left ventricular end diastolic volume
LVEF: Left ventricular ejection fraction
LVESd: Left ventricular end systolic dimension
LVESdi: Left ventricular end systolic dimension indexed
LVESV: Left ventricular end systolic dimension
LVH: Left ventricular hypertrophy
LVOT: Left ventricular outflow tract
LVOTd: Left ventricular outflow tract diameter
MA: Mitral annulus
MAVD: Mixed aortic valve disease
MR: Mitral regurgitation
MRI: Magnetic resonance imaging
MV: Mitral valve
PHT: Pressure half time
PISA: Proximal isovelocity surface area
PLAX: Parasternal long axis
PSAX: Parasternal short axis
PW: Pulsed wave Doppler
QAV: Quadricuspid aortic valve
RF: Regurgitant fraction
RJ: Regurgitant jet
RV: Right ventricle
RVol: Regurgitant volume
SoV: Sinus of Valsalva
STJ: Sino-tubular junction
SV: Stroke volume
TOE: Transoesophageal echocardiography
TTE: Transthoracic echocardiography
UAV: Unicuspid aortic valve
VC: Vena contracta
VCA: Vena contracta area
VTI: Velocity time integral
